# Webcam-Based Exergame for Motor Recovery with Physical Assessment via DTW

**DOI:** 10.3390/s26041219

**Published:** 2026-02-13

**Authors:** Norapat Labchurat, Kingkarn Sookhanaphibarn, Worawat Choensawat, Pujana Paliyawan

**Affiliations:** 1 School of Information Technology and Innovation, Bangkok University, Pathum Thani 12120, Thailand; norapat.labc@bumail.net; 2 Center of Specialty Innovation, Bangkok University, Pathum Thani 12120, Thailand; 3 Ritsumeikan University Research Organization, Ritsumeikan University, Ibaraki, Osaka 567-8570, Japan

**Keywords:** exergaming, physical assessment, motor recovery, MediaPipe, motion analysis, rehabilitation, scoring algorithm, remote monitoring

## Abstract

This paper presents RehabHub, a home-based exergaming system that integrates standardized physical assessment directly into gameplay by using a common webcam and MediaPipe for real-time pose estimation. The system quantifies upper-limb movement quality, specifically abduction, shoulder flexion, and elbow flexion based on FMA-UE guidelines, by applying Dynamic Time Warping (DTW) together with a Z-score-based scoring model that relies on data from non-clinical adult participants. A pilot study, which included movements simulated with a 5-kg resistance band, evaluated three feature-extraction methods. The findings indicate that the single-angle method provides the clearest distinction between normal and abnormal movements, particularly for abduction and elbow flexion. In the case of shoulder flexion, the score separation was less distinct because of movement variability and posture-related angle fluctuations, which suggests that further refinement of feature design is needed. The cloud-based platform supports remote monitoring and gives caregivers access to both performance scores and recorded exercise videos. Overall, the results demonstrate the feasibility of a low-cost webcam-based assessment integrated into exergaming, and they highlight important trends for improving abnormal-movement detection in home rehabilitation systems.

## 1. Introduction

Physical disability is a global challenge that limits motor function and independent daily activities. Conditions such as stroke and adhesive capsulitis contribute significantly to physical impairments. Stroke, which affects one in four adults over 25 in their lifetime, often results in motor impairments on one side of the body [1]. Adhesive capsulitis, or frozen shoulder, causes pain and stiffness, progressively restricting shoulder mobility, with a prevalence of 2% to 5% in the general population [2]. Despite the importance of rehabilitation in improving motor function, long-term therapy remains challenging and requires consistent effort to achieve effective recovery outcomes.

Traditional rehabilitation relies on physiotherapy-guided sessions to restore motor function and mobility. However, the high costs and repetitive nature of these exercises often reduce motivation and limit accessibility [3]. To address these challenges, robotic and hardware-based rehabilitation tools, such as robotic gloves and treadmill systems, have been introduced to enhance engagement and automate therapy. While these devices offer valuable benefits, their high cost, need for specialized training, and potential safety risks make them less practical for home use [4]. As a result, there is an increasing demand for rehabilitation solutions that are affordable, accessible, and engaging for home-based settings.

Affordable off-the-shelf gaming systems, such as the Nintendo Wii, Microsoft Kinect, and Sony EyeToy, have become practical alternatives for home-based rehabilitation [5]. These systems utilize motion-tracking technologies, including handheld controllers and image-processing methods, to monitor human movements accurately. By leveraging interactive and engaging media, they offer an accessible solution for improving motor function and encouraging physical activity in rehabilitation programs. However, these systems typically require specialized hardware, which can increase overall costs and may limit accessibility.

Recent advancements in pose estimation and machine learning have greatly enhanced the accuracy and efficiency of movement tracking [6]. As a result, common webcams can now monitor movements on widely available devices such as laptops and smartphones [7]. This technology is compatible with web browsers and various operating systems, eliminating the need for specialized equipment and making rehabilitation more affordable and accessible. However, sustaining long-term recovery engagement and adherence to rehabilitation programs remains a significant challenge.

Exergaming increases motivation and engagement by transforming repetitive rehabilitation exercises into interactive experiences [8]. By incorporating game elements, these systems lower the perceived effort of therapy and encourage consistent participation through clear goals and achievements. The real-time feedback and measurable progress reflected in game scores help maintain engagement and enhance both cognitive and motor skills [9]. However, many exergaming systems primarily focus on in-game scores, which may not fully represent a physical performance or recovery progression.

Fugl-Meyer Assessment for Upper Extremity (FMA-UE) [10] is a standardized assessment provide essential guidelines for analyzing movements and evaluating physical progress during rehabilitation. The FMA-UE widely used in healthcare and physical therapy for its proven reliability and validity in assessing motor function [11]. Its comprehensive framework evaluates multiple aspects of motor recovery, including range of motion (ROM), muscle coordination, reflex activity, and joint synergy. Therefore, FMA-UE serves as a crucial tool for tracking rehabilitation progress and guiding effective therapy interventions.

This paper presents a web-based motor recovery system that combines exergaming with standardized physical assessments to enhance home-based rehabilitation. The system utilizes MediaPipe [7] for real-time pose estimation and evaluates upper-limb movement quality based on FMA-UE exercise guidelines. Performance is quantified using Dynamic Time Warping (DTW), enabling objective comparison of user movements against a baseline. Importantly, the proposed assessment framework requires only normal movement data from healthy subjects to construct the reference baseline, eliminating the need for patient data during system development.

The main contributions of this study are threefold. First, we introduce a webcam-based exergame system that integrates standardized physical assessment directly into gameplay, enabling real-time rehabilitation and evaluation without specialized hardware. Second, we propose a DTW-based scoring method to objectively quantify upper-limb movement quality using only normal-subject data for baseline modeling. Third, we develop a cloud-enabled platform for performance visualization and remote monitoring, and validate the system through a pilot study with 12 participants, demonstrating its ability to distinguish between normal and abnormal movements.

## 2. Related Works

This section reviews the progression of motor recovery methods, from traditional rehabilitation techniques to modern technology-driven approaches. To identify relevant studies, a structured literature search was conducted across major scientific databases, including IEEE Xplore, Scopus, PubMed, and Google Scholar. Similar works were included based on three key criteria: (1) the use of computer vision and standard webcams for pose estimation, which was examined through keywords such as “computer vision,” “webcam pose estimation,” and “MediaPipe,” (2) the application of exergaming to improve patient engagement in home-based settings, identified using terms such as “exergaming” and “home-based rehabilitation,” and (3) the integration of standardized physical assessment tools such as the FMA-UE and DTW for objective evaluation of motor recovery, using search terms including “FMA-UE” and “Dynamic Time Warping.” These selected studies collectively demonstrate how computer vision and exergaming technologies can support accessible home-based rehabilitation, while validated assessment tools provide quantifiable metrics for tracking recovery progress.

### 2.1. Rehabilitation Technologies

Rehabilitation technologies have evolved from traditional therapist-guided exercises to robot-assisted interventions, offering precise movement assistance while reducing manpower requirements. However, robotic devices are often cost-prohibitive, limiting their widespread accessibility. More recently, computer vision-based solutions have revolutionized home-based rehabilitation by enabling movement analysis and pose estimation using readily available webcams. In addition, exergaming has emerged as a motivational tool, combining interactive gameplay with therapeutic exercises to enhance patient engagement.

#### 2.1.1. Traditional and Hardware-Assisted Rehabilitation

Traditional rehabilitation relies on therapist-supervised exercises to restore motor function. Moncion et al. [12] conducted a scoping review demonstrating the benefits of aerobic exercise in improving cardiovascular health and physical mobility for motor recovery. However, optimizing therapy remains a challenge due to the lack of precise intensity measurement tools.

To address this limitation, hardware-assisted rehabilitation has been introduced. For instance, robotic gloves assist in hand function recovery, providing targeted support for finger movements. Thimabut et al. [13] showed that these gloves improve hand functionality and offer quantitative movement assessments. Despite their effectiveness, high costs and accessibility barriers hinder widespread adoption. Furthermore, maintaining motivation during the lengthy recovery process remains a challenge.

#### 2.1.2. Exergaming for Rehabilitation

Exergaming has emerged as a widely adopted approach in rehabilitation with off-the-shelf devices, significantly enhancing patient engagement and motivation during therapy. For instance, Celesti et al. [14] explored the Ski Slalom game, while Robinson et al. [15] used Tightrope Walk—both from Wii Fit™—to integrate balance training and gait assessment. Trombetta et al. [16] introduced Motion Rehab AVE, where users hit a virtual ball to improve upper limb control and balance. However, many of these systems still require therapist supervision, making them less suitable for independent home rehabilitation.

#### 2.1.3. Home-Based Rehabilitation

Home-based rehabilitation, or telerehabilitation, offers a more accessible solution for motor recovery, by allowing patients regain motor functions in their homes and use to the system without supervision. For instance, Shih et al. [17] utilized a Kinect-based game called Obstacle Avoidance, where users move their bodies to avoid obstacles, promoting physical activity and engagement. Similarly, Pekyavas et al. [18] employed Nintendo Wii Bowling in a home rehabilitation setup, demonstrating significant improvements in motor function. While these systems enhance accessibility, they lack remote monitoring features, preventing therapists from tracking patient progress remotely.

To address this, some systems are designed to include remote monitoring capabilities, allowing physiotherapists to track rehabilitation progress over the internet. For instance, Ferraris et al. [19] developed the REHOME system, which utilizes cloud-based data analytics to track rehabilitation progress. Similarly, Allegue et al. [20] introduced the VirTele rehabilitation program, designed for upper-extremity rehabilitation at home, integrating Reacts (Innovative Imaging Technologies Inc., Montreal, QC, Canada) for video conferencing and patient report sharing. However, these systems rely on depth sensor cameras, which are not widely available in typical home environments.

#### 2.1.4. Leveraging Computer Vision

Computer vision enables home-based rehabilitation without requiring specialized hardware, making it accessible via common webcams and even mobile phones. Burke et al. [9] used color detection with webcams identify hand movements, enabling interaction in their exergames, Rabbit Chase and Arrow Attack. Additionally, rehabilitation data could be uploaded via the internet. Similarly, Wang et al. [21] utilized MediaPipe and common webcams to develop Attack the Monster, a balance-training exergame that transmits rehabilitation progress to physiotherapists online. However, these systems lack standardized physical assessments, making it difficult to objectively measure rehabilitation success compared to clinical evaluations.

#### 2.1.5. Physical Assessment Integrated with Exergaming

Building on these advancements, some systems incorporate additional assessment methods to evaluate physical performance post-exergaming. Rosique et al. [22] introduced ExerCam, a vision-based pose detection system that operates directly within a web browser. ExerCam integrates exergames such as Crazy Target and Boxing, allowing remote monitoring and providing real-time feedback for supervisors. However, this system, along with others such as Shih et al. [17] and Celesti et al. [14], does not perform physical assessment synchronously during exergaming, which may result in longer rehabilitation durations. Therefore, integrating real-time movement data collection or movement analysis within exergaming sessions is crucial for enhancing motivation and reducing rehabilitation time.

#### 2.1.6. Comparison with Existing Exergaming Systems

Table 1 compares our proposed system with existing exergaming-based rehabilitation systems across five key dimensions as follows.

- Requires Only Common Webcam: Determines whether the system works with common webcams (RGB cameras) without requiring specialized hardware.
- Independence from Supervision: Evaluates if patients can undergo rehabilitation without constant therapist oversight.
- Remote Monitoring Capability: Assesses whether caregivers can track rehabilitation progress remotely.
- Integration of Standardized Physical Assessments: Examines if the system includes clinically standardized tools for objective movement analysis.
- Movement Data Collection or Analysis During Exergaming: Identifies if the system records or analyzes movement data in real-time while exergaming.

### 2.2. Standardized Physical Assessments Integrated with Exergaming

Standardized Physical Assessments are clinical tools for evaluating the progress of therapeutic patients, providing objective measures of motor recovery. The FMA-UE offers a comprehensive evaluation of motor impairments, reflexes, and ROM, making it the gold standard in motor recovery. Other assessments, such as the Wolf Motor Function Test (WMFT) [23] and Box and Block Test (BBT) [24], focus on functional task performance and manual dexterity, respectively. Although WMFT and BBT are valuable for specific functions, the FMA-UE’s detailed approach provides deeper insights into impairment-level recovery.

While many exergaming systems rely solely on in-game scores to motivate patients and measure progress, these scores do not always correlate with true physical performance [25]. Some researchers integrated clinical tools for physical assessment, they often evaluate performance separately from exergaming. For example, Allegue et al. [20] conducted FMA-UE assessments before and after exergaming sessions to measure improvements in FMA-UE scores. However, separating physical assessment from exergaming can increase the overall time commitment for patients and workload.

To address this limitation, we propose a system that collects movement data during exergaming. By utilizing the FMA-UE and DTW to provide a comprehensive evaluation of the patient’s physical capabilities. This approach eliminates the need for separate clinical assessments alongside exergaming, which reduces time consumption and streamlines the rehabilitation process.

#### 2.2.1. Fugl-Mayer Assessment Upper Extremity (FMA-UE)

The FMA-UE introduced by Fugl-Meyer et al. in 1975 [10], is a standardized and widely used framework for assessing motor recovery in therapeutic patient. It provides a quantitative evaluation of joint motion, reflex activity, coordination, and voluntary movement, offering a reliable measure of functional recovery. Its effectiveness and validity have been well-established in studies such as Sanford et al. [26], making it a cornerstone tool in both clinical practice and rehabilitation research.

In this study, three FMA-UE exercise poses are used: abduction, shoulder flexion, and elbow flexion.

#### 2.2.2. Dynamic Time Warping (DTW)

DTW is a widely used algorithm in time-series analysis that measures the similarity between temporal sequences with varying speeds, durations, or timings. It is particularly effective in motion analysis and rehabilitation by aligning sequences non-linearly to account for temporal discrepancies, ensuring accurate comparisons of patient movements with normal individuals [27]. In our system, DTW is employed to assess whether a patient’s movement within a motion segment (a single motion performance) aligns with expected patterns, providing precise performance evaluation.

Several studies have leveraged DTW to enhance motion analysis across different domains. Choensawat et al. [28] improved motion capture database retrieval by utilizing DTW to analyze speed patterns, enhancing query efficiency and search effectiveness. Schneider et al. [29] integrated DTW with OpenPose for gesture recognition in video data, enabling a robust and flexible human pose classification system. Yu et al. [27] applied DTW in a Kinect-based rehabilitation system to validate patient exercises by comparing their movements against reference trajectories, ensuring accurate exercise performance assessment.

## 3. The Proposed System

**Figure 1:**
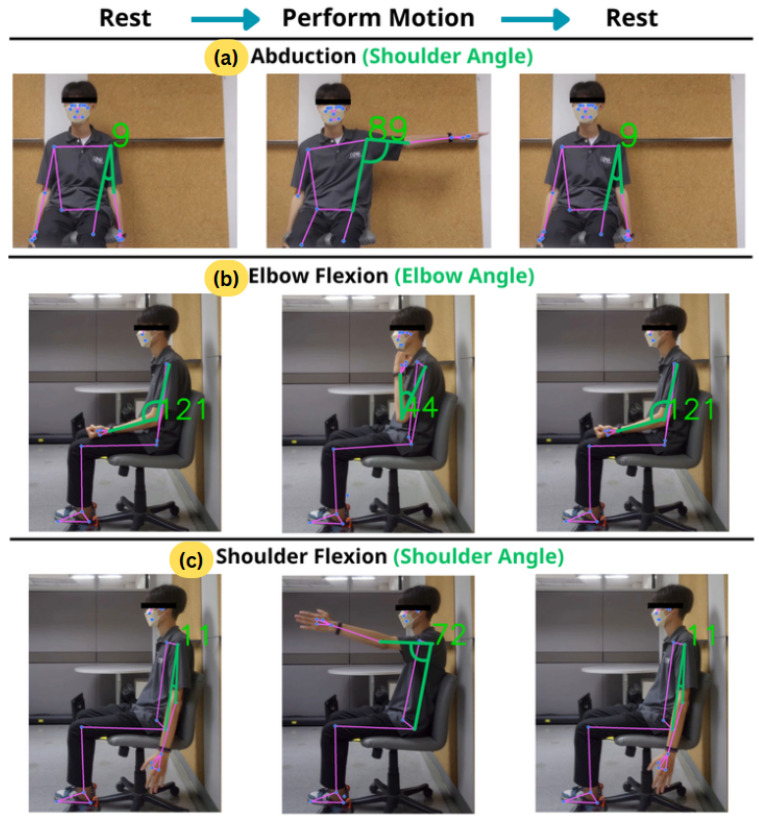
Exercise motions derived from the FMA-UE with range of motion (ROM) detected using the proposed motion detection module (MDM): (**a**) abduction, (**b**) elbow flexion, (**c**) shoulder flexion.

We propose RehabHub (Bangkok University Rehabilitation Hub), a web-based application for upper-limb motor recovery that integrates game-based exercises with standardized physical assessments. In our pilot study, we focused on three fundamental postures from the FMA-UE framework: abduction, shoulder flexion, and elbow flexion, as shown in Figure 1. These postures were selected because they represent essential upper-limb movements frequently emphasized in early rehabilitation, making them ideal for evaluating the system’s motion-tracking and feedback capabilities.

### 3.1. System Overview

**Figure 2:**
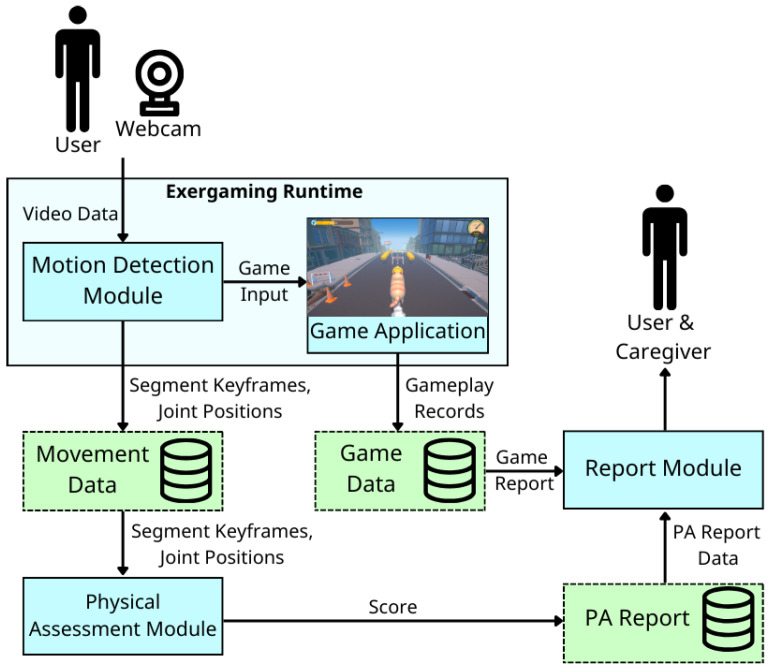
Overview of the proposed system.

The system has four major components (cf. blue boxes in Figure 2). The Motion Detection Module (MDM) receives raw movement data (pose landmarks) via MediaPipe. It then extracts a primary single-joint angle relevant to the exercise and uses this angle to detect motion and control the game character. A single-joint feature is used here because the goal is to capture basic movement intent rather than enforce strict form, ensuring responsiveness and maintaining motivation for patients with limited accuracy or range of motion. The MDM also stores all movement data for later physical assessment.

Details about the functionality and computational processes of each major component are provided in Section 3.2, Section 3.3, Section 3.4 and Section 3.5.

### 3.2. Motion Detection Module (MDM)

The Motion Detection Module (MDM) receives raw movement data (pose landmarks) via MediaPipe. It then extracts the primary single-joint angle (single-joint feature recognition) relevant to the current exercise. This single-angle information is used primarily to detect motion and generate real-time game input, focusing on movement intent over strict form. This flexibility is essential for maintaining user motivation and engagement for patients who may not be able to perform movements with high accuracy or high range of motion. Additionally, it stores the data for further physical assessment.

**Figure 3:**
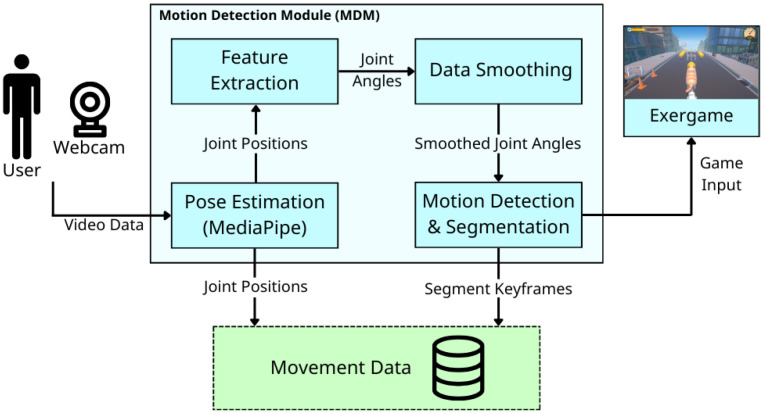
Overview of the Motion Detection Module (MDM). Each box represents a processing step, while arrows indicate the flow of output data between processes.

As shown in Figure 3, MDM consists of four distinct processes, each detailed in the subsequent subsections.

#### 3.2.1. Pose Estimation

**Figure 4:**
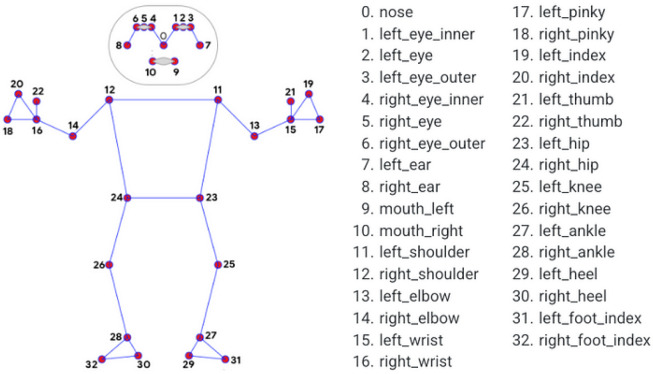
The 33 pose landmarks detected by MediaPipe.

Our system leverages MediaPipe for pose estimation, extracting joint positions from common webcams. MediaPipe is a computer vision and machine learning framework compatible with various devices and platforms, including mobile phones, laptops, tablets, and web applications. It performs real-time pose estimation by detecting 33 human body landmarks, including key joints such as the shoulders, elbows, and wrists, as shown in Figure 4. This functionality allows our system to use only common webcams for joint extraction, ensuring broad compatibility with commonly available home-use devices.

In MediaPipe, *x* and *y* represent the normalized horizontal and vertical positions of landmarks (joints), scaled between 0 and 1 relative to the image’s width and height. The *z*-coordinate, which estimates the relative depth of landmarks from the camera, is less reliable as it is derived from pose estimation rather than actual depth sensing, and does not provide a true 3D measurement [30]. Consequently, our system utilizes only the *x* and *y* coordinates for motion analysis, excluding the *z*-axis from this study.

#### 3.2.2. Feature Extraction for Motion Detection

Joint angles are computed using the Cartesian coordinates of three key points: the vertex point $\left(x_{v},y_{v}\right)$, which represents the joint of interest, and two additional points $\left(x_{1},y_{1}\right)$ and $\left(x_{2},y_{2}\right)$, which define the vectors extending from the vertex. The angle at the vertex is then determined using the Cosine Rule (Law of Cosines), which relates the dot product of these vectors to the angle between them. The angle at the vertex point is obtained using this relationship, as expressed in the following formula:

(1) $$D=\left(x_{1}-x_{v}\right)\left(x_{2}-x_{v}\right)+\left(y_{1}-y_{v}\right)\left(y_{2}-y_{v}\right)$$

(2) $$M_{1}=\sqrt{{\left(x_{1}-x_{v}\right)}^{2}+{\left(y_{1}-y_{v}\right)}^{2}}$$

(3) $$M_{2}=\sqrt{{\left(x_{2}-x_{v}\right)}^{2}+{\left(y_{2}-y_{v}\right)}^{2}}$$

(4) $$J=\arccos\left(\frac{D}{M_{1}\cdot M_{2}}\right)\cdot \frac{180}{\pi }$$

where the dot product (*D*) of the vectors is computed using Equation (1), while the magnitudes of the vectors are given by $M_{1}$ and $M_{2}$ in Equation (2) and Equation (3), respectively. *J* represents the joint angle in degrees, calculated by applying Equation (4).

This methodology adapts to various postures by selecting a specific joint angle for each motion. For example, elbow flexion is measured using the angle at the elbow—computed from the vectors defined by the shoulder and wrist. Similarly, shoulder movements are quantified using the angle at the shoulder, determined by the vectors from the elbow and hip. Notably, both abduction and shoulder flexion use this same joint angle measurement, but they require different camera orientations for accurate capture.

Proper webcam positioning is crucial for clear joint movement visibility. For abduction, the webcam should be placed in front of the user, as shown in Figure 1a, whereas for shoulder and elbow flexion it should be positioned to the side, as illustrated in Figure 1b,c. Recognizing that physically repositioning a fixed webcam may be impractical for home users, the simplest solution is for the user to utilize a rotatable chair or adjust their seating position to ensure proper alignment with the camera’s view. Aligning the webcam with the movement direction is essential for accurate 2D pose estimation; angle measures, such as the Cosine Rule applied here, are inherently susceptible to deviations caused by camera perspective or occlusion, strongly emphasizing the importance of precise setup for minimizing error [31].

#### 3.2.3. Data Smoothing for Motion Detection

The moving average technique [32] is applied to smooth angle data and reduce noise in pose estimation. This method minimizes fluctuations caused by random errors or measurement inconsistencies, resulting in a clearer and more stable motion representation. Empirical testing determined an optimal window size of 5, effectively balancing noise reduction with trajectory preservation. By averaging data points within this window, the filter smooths short-term variations while maintaining overall motion trends.

#### 3.2.4. Motion Detection and Segmentation

**Figure 5:**
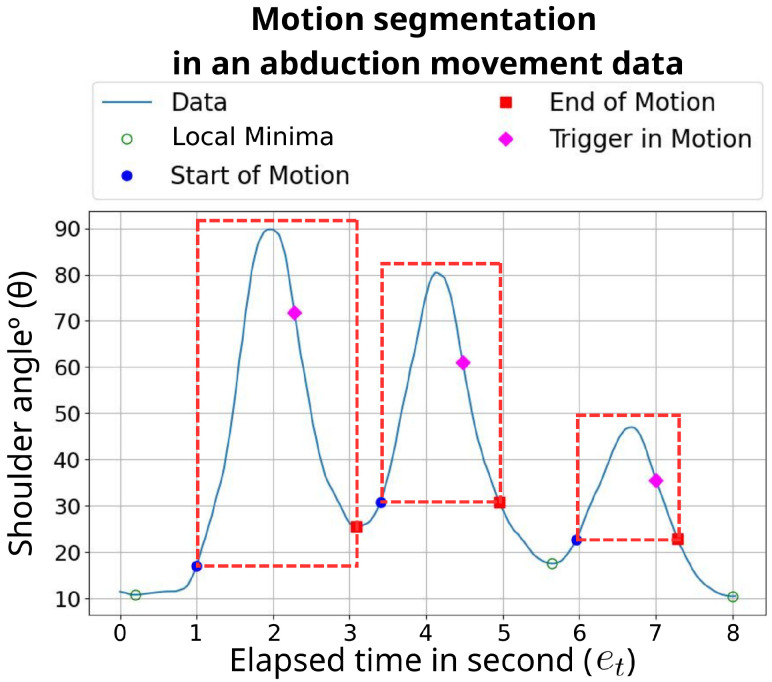
An example of motion segmentation from movement data, demonstrating the adaptability of our algorithm to various levels of impairment. The blue circle represents the start of a motion segment, while the red square marks the end. Each red box outlines a motion segment within the movement, showcasing the algorithm’s dynamic capability in detecting and segmenting diverse motion patterns.

This process utilizes the joint angle at the vertex point of the selected exercise motion, as illustrated in Figure 5, to detect and segment continuous movement into distinct motion segments. During exergaming, the user selects an exercise motion, which defines the vertex point. This point is used in conjunction with a trigger input detection algorithm from our previous work [33] and the publicly available implementation in [34] to identify local extrema and segment the motion.

The system detects when the joint angle deviates from the rest position, marking the Start of Motion. It then identifies the moment when the angle begins returning toward the rest position (i.e., just after the movement peak), which is defined as the trigger point. This trigger point (the magenta diamond in Figure 5) is used primarily to trigger game input events.

To determine the ending frame, if the angle returns to the same value as the starting position, that frame is marked as the end of the segment. If the angle does not return (e.g., because the motion immediately reverses for the next segment), the local extremum, either a maximum or minimum depending on the joint and movement, is used as the end, as illustrated in the first motion segment in Figure 5. The resulting motion segment (red dashed box in Figure 5) spans from the Start of Motion to this determined end point. By combining trigger detection with return-to-start and extremum analysis, the system reliably segments continuous movements into distinct analyzable segments.

### 3.3. Physical Assessment Module (PAM)

The Physical Assessment Module (PAM) analyzes movement in a given motion segment and provides a score indicating how closely it matches a target movement performed by average normal individuals. Unlike MDM, which relies on single-joint dominant features to characterize motion identity, the PAM uses multi-joint, multi-angle trajectories because accurate assessment requires recognizing compensatory patterns and evaluating overall movement quality. Each motion segment is represented by these joint-angle trajectories and compared with mean trajectories obtained from a normal subject sample using Multi-dimensional DTW. The resulting DTW distance is then converted into a similarity score on a scale of 0 to 100.

**Figure 6:**
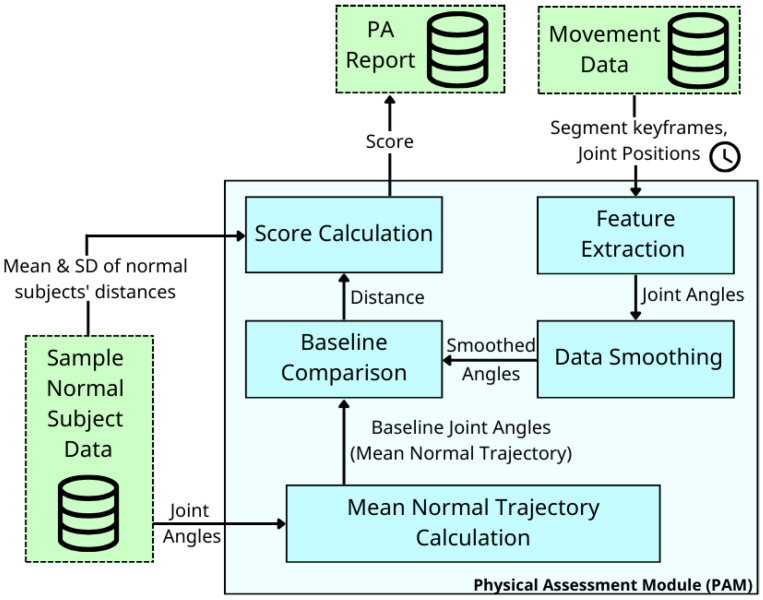
Overview of the Physical Assessment Module (PAM). Each blue box represents a processing step, while arrows indicate the flow of output data between processes.

As shown in Figure 6, the Physical Assessment Module (PAM) consists of five distinct processes, each detailed in subsequent subsections.

#### 3.3.1. Feature Extraction for Physical Assessment

The Physical Assessment Module (PAM) extends the feature extraction approach used in the MDM framework. Feature extraction can be performed using different sets of joint angles, allowing flexibility in analyzing movement characteristics. In this study, one example method involves using three joint angles—shoulder, elbow, and wrist—to assess exercise motions.

This multi-angle approach is particularly important for detecting movement compensations. Individuals with physical impairments may struggle to perform certain movements correctly, often compensating by engaging other body parts. For instance, a subject experiencing difficulty with elbow flexion may unintentionally open their shoulder to assist the motion.

During feature extraction, raw joint position data is retrieved from the database and segmented into distinct motion phases based on keyframes identified during segmentation. The selected joint angles are then computed. In the case of wrist angle calculation, the wrist serves as the vertex, while the index finger and elbow define the vectors. Ultimately, each motion segment is represented by a set of joint angle trajectories.

The optimal set of joint angles for feature extraction will be evaluated in the experiment section, comparing single-angle and multi-angle approaches to determine the most effective representation for movement assessment.

#### 3.3.2. Data Smoothing for Physical Assessment

Similar to the approach used in MDM, the extracted joint angles in PAM undergo a smoothing process to reduce noise and enhance the clarity of motion trajectories.

#### 3.3.3. Mean Normal Trajectory Calculation

**Figure 7:**
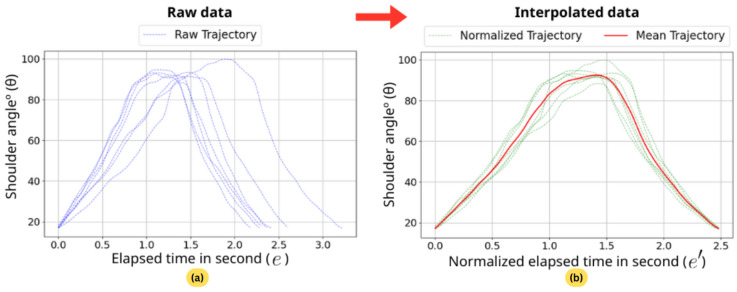
Visualization of the mean trajectory calculation process. (**a**) Raw trajectory data from individual motion segments of normal sample subjects. (**b**) Raw trajectories normalized to the mean length (dotted green lines), with the resulting mean normal trajectory represented by the solid red line.

The system requires a sample set of normal movement data as a reference. Trajectories from normal motion segments are analyzed to establish a baseline, which is determined through averaging and interpolation, as illustrated in Figure 7. Specifically, independent trajectories from each motion segment are averaged to derive the mean normal trajectory for each joint angle, serving as a standardized reference for evaluating motion deviations.

To compute the mean normal trajectory of a joint angle, the mean trajectory length is first determined. Since the length of a trajectory is essentially the maximum elapsed time ($l=e_{\max}$), the mean length is calculated as follows:

(5) $$\bar{l}=\frac{\sum_{i=1}^{n}l_{i}}{n}$$

where $l_{i}$ represents the length, specifically the maximum elapsed time of the *i*-th trajectory. Here, *n* is the total number of trajectories, *i* denotes the trajectory index, and $\bar{l}$ represents the mean length.

The next step is to normalize the sample normal trajectories to a standardized length, effectively stretching or compressing each trajectory to match the mean length. This process transforms the blue lines into green lines in Figure 7. Once normalized, the elapsed time for each data point is adjusted from the original elapsed time *e* to the new elapsed time $e^{\prime }$. This time transformation for each single trajectory is computed using the following equation:

(6) $$e_{t}^{\prime }=e_{t}\cdot \frac{\bar{l}}{e_{\max}}$$

where $e_{t}^{\prime }$ is the new elapsed time, $e_{t}$ is the original elapsed time, $\bar{l}$ is the mean length of trajectories, and $e_{\max}$ is the length of the trajectory of interest.

After normalization, the angle value at any elapsed time *t* on the normalized trajectory can be estimated using linear interpolation as follows:

(7) $$\theta_{t}=\theta_{b}+\left(e_{t}^{\prime }-e_{b}^{\prime }\right)\cdot \frac{\theta_{a}-\theta_{b}}{e_{a}^{\prime }-e_{b}^{\prime }}$$

where $\theta_{t}$ is the interpolated angle value at elapsed time *t*, $\theta_{b}$ and $\theta_{a}$ are the angle values at the nearest preceding and subsequent data points, respectively. The variables $e_{b}^{\prime }$ and $e_{a}^{\prime }$ represent the elapsed times corresponding to $\theta_{b}$ and $\theta_{a}$, while $e_{t}^{\prime }$ is the elapsed time of the targeted interpolated data point.

Once the angle values at any elapsed time are accessible for all trajectories, the average angle $\bar{\theta }$ is computed across all trajectories using the following equation:

(8) $${\bar{\theta }}_{t}=\frac{1}{n}\sum_{i=1}^{n}\theta_{t,i}$$

where ${\bar{\theta }}_{t}$ represents the mean angle value at elapsed time *t*, *n* is the total number of trajectories, and $\theta_{t,i}$ denotes the angle value of the *i*-th trajectory at elapsed time *t*.

The result of averaging the angles across all trajectories is a set of mean angles, also referred to as the mean trajectory for a single joint (the red line in Figure 7), denoted as *M*, and defined as follows:

(9) $$M=\{{\bar{\theta }}_{0},{\bar{\theta }}_{1},{\bar{\theta }}_{2},\ldots ,{\bar{\theta }}_{\bar{l}}\}$$

Finally, the baseline for evaluating a motion segment is established by combining the mean trajectories of the three joint angles. This baseline, denoted as *B*, represents the mean normal trajectories and is defined as follows:

(10) $$B=\left[\begin{array}{c} M_{\mathrm{shoulder}}\\ M_{\mathrm{elbow}}\\ M_{\mathrm{wrist}} \end{array}\right]$$

where $M_{\mathrm{shoulder}}$, $M_{\mathrm{elbow}}$, and $M_{\mathrm{wrist}}$ correspond to the mean trajectories of the shoulder, elbow, and wrist angles, respectively.

#### 3.3.4. Baseline Comparison

Baseline comparison is conducted using multi-dimensional dynamic time warping (Multi-DTW), which compensates for temporal variations across multiple joint angles within a motion. This method measures the similarity between a given motion segment α and the normal baseline *B* established in the previous section. The resulting distance metric quantifies the degree of deviation from the normal baseline, providing an indicator of motion abnormality.

The difference between the segment of interest and the baseline is evaluated using Multi-DTW, defined as:

(11) $$d_{\alpha }=\mathrm{Multi-DTW}\left(\alpha ,B\right)$$

where $d_{\alpha }$ represents the dissimilarity as a distance between the assessed motion segment and the normal baseline *B*.

#### 3.3.5. Score Calculation

The distance $d_{\alpha }$ is assigned to a scale of 0 to 100, where higher scores represent closer alignment to normal movement. The system is designed to calculate these scores using only sample data from normal subjects, without the need for data from patients. This calculation leverages statistical analysis based on the normal distribution, implemented through a piecewise function.

First, $d_{\alpha }$ is normalized using the Z-score formula, which is based on the mean and standard deviation (SD) of the distances from normal sample subjects. The Z-score is calculated as follows:

(12) $$Z_{\alpha }=\frac{d_{\alpha }-\mu }{\sigma }$$

where $Z_{\alpha }$ is the Z-score of the segment being assessed, $d_{\alpha }$ is the distance of the current motion segment, μ is the mean distance of the normal sample data, and σ is the SD of the normal sample distances. This normalization enables comparisons across different subjects and ensures the distance measure is standardized with respect to the normal movement data.

The Z-score is then used to calculate the score $S_{\alpha }$ according to the following rules:

(13) $$S_{\alpha }=\left\{\begin{array}{cc} 100 & \mathrm{if}\;Z_{\alpha }\leq 1\\ \frac{100}{Z_{\alpha }} & \mathrm{if}\;Z_{\alpha }>1 \end{array}\right.$$

which can be simplified as:

(14) $$S_{\alpha }=\frac{100}{\max(Z_{\alpha },1)}$$

**Figure 8:**
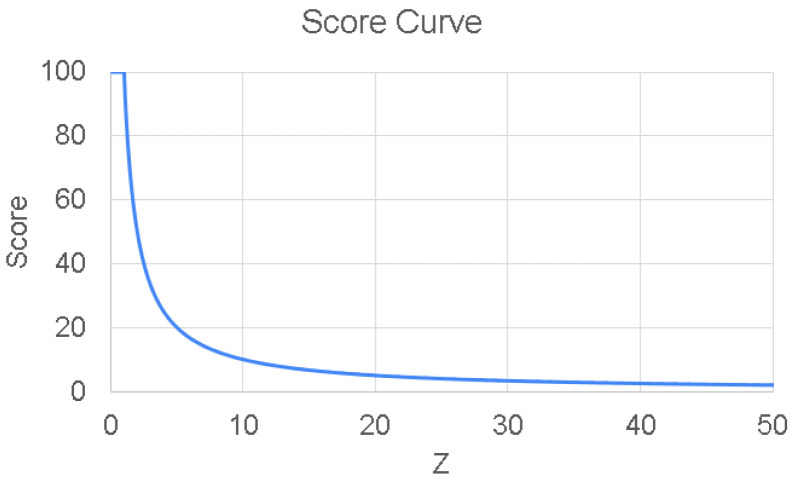
Mapping of Z-scores to corresponding scores used in the system.

This scoring system ensures that higher Z-scores ($Z_{\alpha }$), which indicate greater deviation from the baseline, result in lower scores. A perfect score is obtained when the distance is within one SD from the mean of the normal subject data. The relationship between the Z-score and the calculated score is illustrated in Figure 8.

### 3.4. Exergame

The exergaming component of our system consists of two major elements: (1) A Pilot Exergame: Meow Runner, which serves as a prototype for motion-based gameplay, and (2) Motion to Game Input, which establishes a plugin to facilitate communication between the exergame and MDM through a web browser, demonstrating the capability of utilizing motion-based inputs in Meow Runner.

#### 3.4.1. A Pilot Exergame: Meow Runner

**Figure 9:**
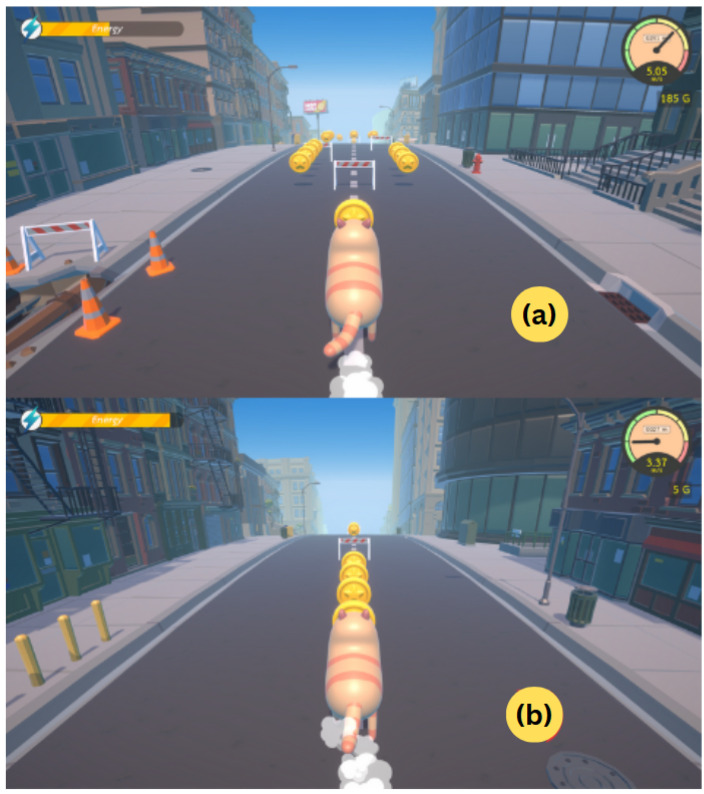
A pilot exergame: Meow Runner. (**a**) Two-Side mode featuring three lanes, allowing movement across multiple directions. (**b**) One-Side mode with a single lane, focusing on jump-based control.

Meow Runner is a 3D runner game designed for therapeutic rehabilitation, as illustrated in Figure 9. Users control a cat running through a city, with speed gradually increasing over time. The game environment consists of three lanes in the Two-Side mode (Figure 9a) and a single lane in the One-Side mode (Figure 9b). The game features procedurally generated obstacles and environments, allowing users to adjust difficulty levels based on their abilities and rehabilitation needs. Our exergame enables users to engage in gameplay by performing postures and offers difficulty selection to adjust the gameplay according to their abilities.

Unlike traditional games that end upon collision or incorrect actions, Meow Runner introduces a forgiving mechanic where collisions with obstacles slow the player down instead of causing a game-over. The game’s objectives are to avoid obstacles, collect coins, and cover the maximum possible distance within a three-minute session. This design encourages users to engage with the game without the fear of failure.

Further difficulty adjustment can be achieved through Dynamic Difficulty Adjustment (DDA), which allows real-time modification of game difficulty. In Meow Runner, the game reduces the character’s speed upon collision, while successful dodges or collecting multiple coins increase the speed. Additionally, physical performance, such as user movement speed, can further fine-tune the game difficulty to match individual capabilities. The implementation of DDA is planned for future work, enabling the game to dynamically adjust difficulty in real-time based on both the user’s in-game performance and physical performance.

#### 3.4.2. Motion to Game Input

We developed a game plugin that provides a high-level interface and functionality for receiving input commands and events from the MDM. This plugin functions as a virtual input device, allowing exergame developers to handle input commands similarly to keyboard key-code functions. Communication between the MDM and our web-based exergame system occurs in real-time using React-Unity-WebGL, which offers APIs and functionalities for two-way communication without requiring network transmission. Consequently, our plugin directly receives game input from the MDM, enabling our system to support external exergames.

For Meow Runner, the game input consists of two modes: Two-Side mode, which uses both sides of the body, and One-Side mode, which relies on a single side. The system was successfully tested using the three fundamental FMA-UE postures selected in the pilot study (abduction, shoulder flexion, and elbow flexion) as motion inputs for controlling the game. In Two-Side mode, motions on one side of the body switch the cat between lanes, while simultaneous motions on both sides trigger a jump to avoid obstacles. In One-Side mode, motions performed on a single side directly trigger a jump. These two modes allow flexible exercise motion design while accommodating different gameplay styles and sides of movement.

### 3.5. Report Module

The Report Module in RehabHub is a statistics page for presenting user performance, covering both in-game and physical performance. It consists of three main pages designed to visualize rehabilitation progress. The first page is the dashboard, which summarizes the progress of the user, such as improvements in physical scores and duration of exercise in the last three days. The second page is the table page, which displays a chronological record of rehabilitation sessions, where each session (e.g., three minutes per session in Meow Runner) is logged for detailed tracking. The last page is the detail page for an individual session, which presents an angle line graph of movements and the physical score for each motion performed during the session. It also includes a recorded video of the user, along with in-game scores and gameplay performance. This module provides a visualization of user performance, integrating physical assessment and in-game data, while recorded videos enable further analysis of physiotherapy analysis.

### 3.6. RehabHub Web Application

**Figure 10:**
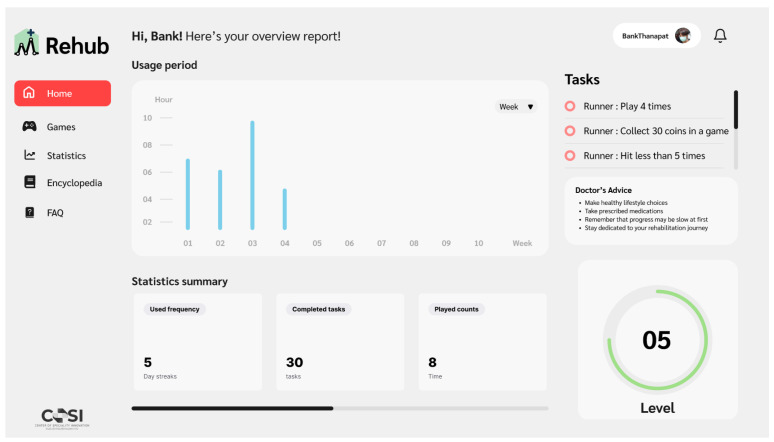
Overview of the RehabHub web application.

We have developed a web application for therapeutic rehabilitation named *RehabHub* (see Figure 10), designed to provide patients with access to game-based rehabilitation programs while allowing physicians to remotely monitor progress and adjust therapy plans. The web-based platform ensures accessibility on any device with a browser and webcam, offering flexibility to manage rehabilitation from home.

The application is organized into six primary tabs:

- Dashboard: Summarizes the patient’s progress, displaying metrics such as completed exercises and performance scores.
- Task Bar: Lists prescribed activities and exercises assigned by doctors.
- Doctor’s Advice: Features personalized guidance and instructions tailored to the patient’s progress and needs.
- Game Library: Provides access to rehabilitation programs, including exergames that utilize postures for interactive inputs.
- Statistics: Provides detailed visualizations of patient performance, including recorded videos and progress metrics across rehabilitation activities.
- Encyclopedia and FAQ: Offers educational content about physical disability and answers to common questions about rehabilitation and the application.

RehabHub enabling doctors to remotely monitor patients through recorded exercise videos and detailed metrics, such as activity trends and physical scores. Doctors can provide real-time feedback and adjust therapy plans to meet individual patient needs. For patients, the platform ensures easy access to exercises and offers visual progress tracking, encouraging active participation in therapy from home. RehabHub promotes engagement and effectiveness for both patients and doctors, ultimately contributing to improved rehabilitation outcomes.

## 4. Experiment

This experiment aimed to evaluate the effectiveness of the proposed DTW-based approach in classifying normal and abnormal movements. The evaluation focused on three key aspects:

1. Assessing the system’s ability to differentiate between normal and abnormal movements.
2. Identifying the optimal feature extraction method for representing motion segments, such as using a single-angle versus multiple angles.
3. Exploring the scoring system, which quantifies motor performance based solely on normal training samples, applied to test samples of both normal and abnormal movements.

### 4.1. Data Collection

#### 4.1.1. Participants

The data collection took place at the Center for Specialty and Innovation (CoSI) Lab, Bangkok University (BU), Thailand. A total of 12 healthy adult male university students (aged 19–23 years) participated in the study. All participants self-reported good health, provided their written informed consent after reviewing the study procedures, and participated voluntarily.

The sample included individuals with varying heights and weights. To maintain consistency in appearance, participants were required to wear a university polo shirt during the data collection process.

#### 4.1.2. Apparatus

During the data collection, video recordings were made of each participant performing exercise motions using a Samsung Galaxy S22+ mobile phone (Samsung Electronics Co., Ltd., Suwon, Republic of Korea). The device’s selfie camera captured the videos at a resolution of 1080 × 1920 (vertical) with a 9:16 aspect ratio, at 60 FPS in full HD H.264 AAC format. The phone was stabilized using a holder placed on top of a chair, while the participant performed the exercises seated on another chair positioned approximately 3 m away. A white projector screen was used as a background to provide a clear and uniform visual reference.

**Figure 11:**
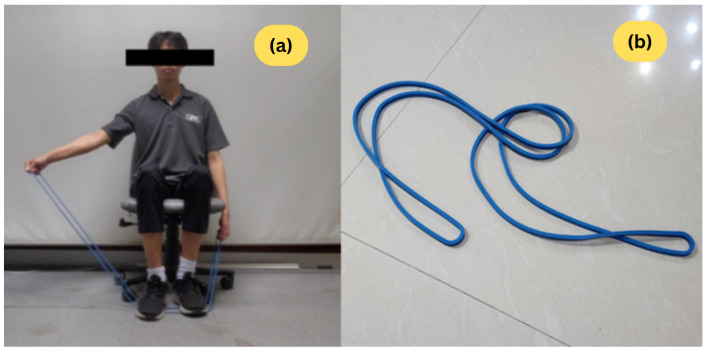
(**a**) A participant performing the abduction exercise motion using a 5-kg resistance band to simulate abnormal movement data. (**b**) The 5-kg resistance band used in the experiment.

In addition, We utilized a 5-kg resistance band to simulate abnormal movements by introducing quantifiable constraints and resistance (see Figure 11). The 5-kg resistance band was utilized to simulate movement constraints, mimicking abnormal movements commonly associated with certain physical disabilities. This simulation method aligns with established research that shows elastic resistance bands reliably increase target muscle forces and reduce the ROM in healthy subjects [35], providing a reasonable foundation for generating the measurable deviations and constrained movement patterns necessary for validating our Dynamic Time Warping (DTW) assessment algorithm.

#### 4.1.3. Procedure

Each participant first watched a 3-min and 12-s instructional video explaining the study procedure, the required movements, and how their data would be used. The video also outlined the study’s purpose and potential risks to ensure participants had a clear understanding. After viewing the video, participants were given the option to participate. Those who agreed signed a consent form detailing their rights, including voluntary participation, confidentiality, and the ability to withdraw at any time without penalty.

Upon providing consent, participants performed three specific exercise motions: elbow flexion, shoulder flexion, and shoulder abduction, separately for each side of the body (see Figure 1). For each motion and side, participants completed two rounds of normal movement and one round of abnormal movement. In each round, they performed the motion 10 times while an observer counted aloud. In total, each participant completed 180 motion repetitions (equivalently, segments)—120 normal and 60 abnormal—across all motions and sides.

To minimize fatigue and maintain consistent performance, rest periods were provided between rounds. A 30-s rest was given between rounds of the same motion and side, allowing brief recovery before continuing. A one-minute rest was provided when switching to a different motion or side, ensuring participants had sufficient time to reset and prepare.

#### 4.1.4. Movement Execution Protocol

For normal movements, participants performed each motion naturally, avoiding excessive force or slowness while ensuring they reached the full range of motion. The goal was to replicate typical, smooth movement to establish a baseline for motor performance. Each repetition lasted approximately one second, with participants instructed to maintain steady, controlled motion throughout.

For abnormal movements, participants used a 5-kg resistance band by stepping on it with both feet to ensure equal band length on both sides while holding it with both hands, as illustrated in Figure 11. In this condition, participants were not required to reach their full range of motion. Instead, they performed the movement slowly and carefully, similar to weight training, which could induce slight muscle fatigue and shaking. Each repetition took approximately two to three seconds.

#### 4.1.5. Recorded Data

Each recording clip captured one round of a performed motion. The recording process began with the observer pressing the record button, followed by a countdown from three before the participant started moving. After completing 10 repetitions, the participant held the final position for approximately two seconds before stopping the recording.

A total of 216 video clips were collected from 12 participants. Each participant contributed clips of both normal and abnormal movements for different motions and sides of the body. These recorded videos will be used for further analysis.

### 4.2. Analysis and Results

#### 4.2.1. Dataset Preparation

All video clips were processed using MediaPipe, with movements segmented into individual motion segments. Each clip yielded 10 motion segments, resulting in a total of 2160 segmented motions. After feature extraction, data from both left and right sides were combined, leading to 720 motion segments per exercise motion, consisted of 480 normal movements and 240 abnormal movements.

To facilitate analysis, the 720 motion segments for each exercise were evenly divided into three subsets:

- Baseline Normal Movement (Subset B)—Normal data used for training, forming the mean normal trajectories, and consequently the baseline (referred to as “Sample Normal Subject Data” in Figure 5).
- Test Normal Movement (Subset N)—Normal data used for evaluating the system’s performance.
- Test Abnormal Movement (Subset A)—Abnormal data used for testing movement differentiation.

The 480 normal motion segments were randomly split between subsets B and N. Subsets N and A were used primarily for system evaluation in this study. Each participant contributed an equal number of motion segments to ensure balanced data distribution.

#### 4.2.2. Analysis of Distance

This analysis focuses on the first two aspects of evaluation, which correspond to evaluating how effectively the system distinguishes between normal and abnormal movements. It also examines how the “Baseline Comparison” process, shown in Figure 5, can be optimized by testing different feature sets.

Three feature extraction methods are considered:

- Single-angle—Using only the main joint angle, specifically the shoulder for abduction and shoulder flexion, and the elbow for elbow flexion.
- Dual-angle—Using a combination of shoulder and elbow angles for every exercise motion.
- Triple-angle—Using three angles, including an additional wrist angle.

**Figure 12:**
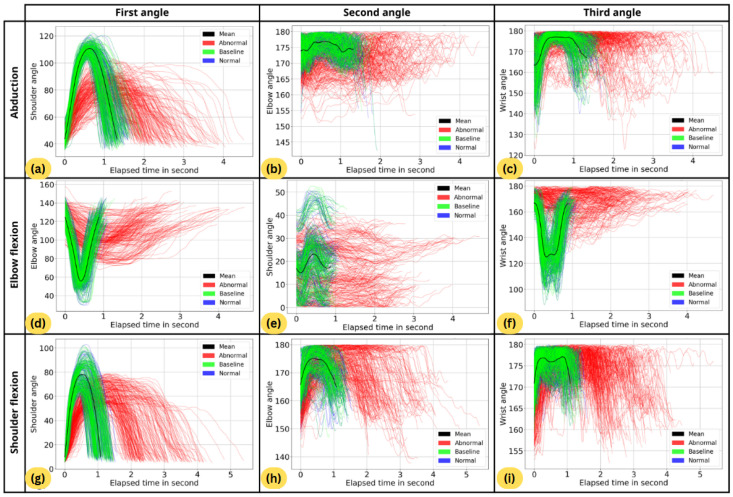
Smoothed angle trajectories from 720 motion segments across three exercise motions and three joint angles. Rows correspond to exercise motions and columns to joint angles. Baseline (green), normal (blue), and abnormal (red) trajectories are shown, with the mean Baseline trajectory in black. (**a**–**c**) Abduction exercise: shoulder (primary), elbow, and wrist angles. (**d**–**f**) Elbow flexion exercise: elbow (primary), shoulder, and wrist angles. (**g**–**i**) Shoulder flexion exercise: shoulder (primary), elbow, and wrist angles.

Figure 12 visualizes the smoothed angle data from 720 motion segments across three exercise motions and three joint angles (referred to as 720 movement trajectories). The figure is organized as a 3×3 grid, where each row corresponds to an exercise motion and each column represents a joint angle. Each plot compares Baseline (green lines), Normal (blue lines), and Abnormal (red lines) data groups, with the mean normal trajectory, constructed from the Baseline data, shown as a black line.

For each exercise motion, trajectories from subset B are used to construct the mean trajectory according to Equation (9). The first column corresponds to the primary joint angle associated with the exercise motion, the second column represents an additional shoulder or elbow angle, and the third column shows the added wrist angle.

The distance between each motion segment in all three subsets and the baseline is then computed. The method of distance measurement depends on the feature extraction methods. For the single-angle method, the baseline consists of only one mean trajectory, represented by the black line in the graph. The distance is measured between each of the 720 lines and the black line in the same graph using DTW. For the Dual and triple-angle methods, the distance is computed by applying DTW across a combination of angles. In the case of the triple-angle method, the baseline is a combination of three black lines, each representing the mean trajectory for one angle, as discussed in Equation (10), and the distance is computed across all three angles.

We then analyzed whether there were a difference in the distance between different groups (subsets). The expected result was that there is no significant difference between subsets B and N, as both represent normal movements, while there were a difference between B and A, as well as between N and A. The analysis was conducted using the Independent Samples *t*-test and the Mann-Whitney U-test. The results are shown in Table 2 and Table 3.

The findings based on the above tables are as follows.

- The single-angle method provides the expected results in all cases under both tests. It can be observed that the shoulder angle effectively distinguishes between normal and abnormal movement for abduction and shoulder flexion, with a *p*-value less than 0.0001. The elbow angle yields similar results when used for elbow flexion.
- The triple-angle method provides good results only for abduction and elbow flexion. However, for shoulder flexion, it was found that the two normal data subsets, B and N, are significantly different. Since the mean trajectory is calculated from subset B, it is possible that the mean normal trajectories overfit the training data.
- For shoulder flexion, there are no significant differences in elbow angle between all pairs of subsets. Based on the U-test, the *p*-values between normal (subsets B or N) data and abnormal (subset A) are 0.9745 and 0.0854, respectively. These *p*-values are notably different, indicating that this feature may be sensitive to sample data and could contribute to the issue mentioned above. We suggest that the elbow angle be omitted when analyzing shoulder flexion.
- For elbow flexion, based on the *t*-test, the shoulder angle shows no significant difference between subsets B and A, but there is a significant difference between N and A. The *p*-values are close to the 0.05 threshold. Additionally, in Figure 12e, it can be observed that the shoulder angle is vertically dispersed, lacking a uniform pattern. Therefore, the result appears sensitive to statistical coincidence, and, similarly to the previous case, we believe the shoulder angle should be omitted when analyzing elbow flexion.
- Both single-angle and dual-angle methods provide good results in both the *t*-test and U-test. However, since it was concluded that the elbow angle is not recommended for shoulder flexion and the shoulder angle is not recommended for elbow flexion, the dual-angle method, which uses both angles for all motions, is less recommended than the single-angle method, which already performs well.
- The wrist angle alone shows good results in all cases except one (the comparison between subsets B and A for shoulder flexion in the *t*-test). However, we later realized that the wrist angle might be affected because participants simulated abnormal movement data by holding a 5-kg resistance band (cf. Figure 11). Furthermore, the range of motion for the wrist is relatively small compared to the main joint angles, meaning that fluctuations in this angle are minimal; if all angles are equally weighted, the wrist angle could be more sensitive to small noise, potentially impacting the overall accuracy. Therefore, the wrist angle should be excluded if the single-angle method already performs well enough.

In summary, the results indicate that the single-angle method is the most reliable for distinguishing between normal and abnormal movements across all exercises. While the dual-angle and triple-angle methods showed some effectiveness, they introduced inconsistencies, particularly in shoulder flexion, likely due to overfitting or sensitivity to sample variations. Additionally, the elbow angle was found to be unreliable for shoulder flexion analysis, and the shoulder angle was unreliable for elbow flexion. The wrist angle, although generally effective, may have been influenced by external factors such as the resistance band. Given these findings, the single-angle method emerges as the preferred approach for motion assessment in this system. It not only provides accurate differentiation but also offers a simpler and more efficient solution compared to multi-angle approaches.

#### 4.2.3. Exploration of the Scoring Mechanism

This section addresses the final aspect of evaluation: quantifying motor performance using normal training data and applying the resulting scoring system to test samples containing both normal and abnormal movements. The goal is to assess how effectively the scoring mechanism differentiates movements and reflects deviations from expected motion patterns. By leveraging the mean normal trajectories, we evaluate how these deviations influence scores and explore the reliability of the scoring system in reflecting motor performance.

**Figure 13:**
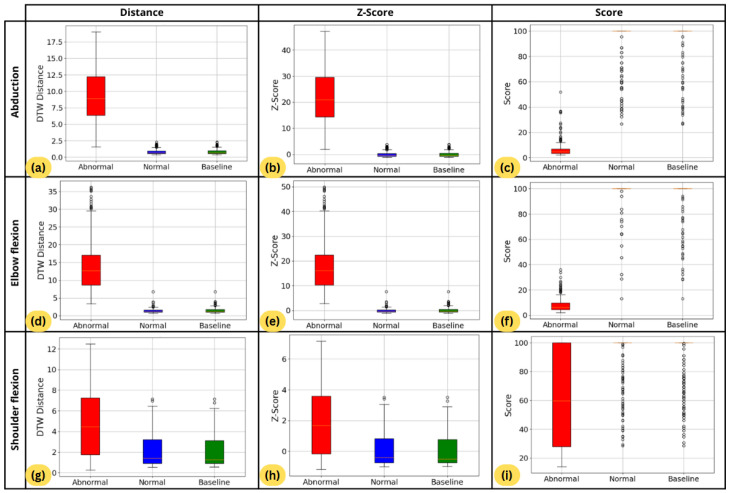
A 3×3 grid of boxplots comparing baseline, normal, and abnormal data groups. (**a**–**c**) Abduction: (**a**) distance to the mean trajectory; (**b**) corresponding Z-scores; (**c**) final scores derived from Z-scores. (**d**–**f**) Elbow flexion: (**d**) distance to the mean trajectory; (**e**) corresponding Z-scores; (**f**) final scores derived from Z-scores. (**g**–**i**) Shoulder flexion: (**g**) distance to the mean trajectory; (**h**) corresponding Z-scores; (**i**) final scores derived from Z-scores.

Figure 13 presents nine boxplots arranged in a 3 × 3 grid. The first column shows the distributions of distances to the mean trajectory, calculated using the single-angle method (Mean and SD values are provided in Table 4). As expected, subsets B and N exhibit similar distributions, indicating comparable normal movements. In contrast, subset A shows significantly higher distances, demonstrating the system’s ability to distinguish abnormal movements. The gap between subset A and the other subsets is particularly pronounced in abduction and elbow flexion. However, for shoulder flexion, overlapping interquartile ranges suggest greater difficulty in differentiating normal and abnormal movements. Notably, in Table 4, the mean distance for Subset A is approximately equal to the mean of B plus one SD, highlighting the reduced separation in this motion.

Next, each distance value is normalized using Z-score normalization, as outlined in Equation (12), with the mean and SD derived from subset B. The normalized results are shown in the second column of Figure 13. According to the empirical rule for normal distribution, data with a Z-score greater than 2 are considered significantly higher than the baseline reference data, corresponding to about a 95% confidence level. Across the three exercise motions, subset A (abnormal data) shows considerable variation, with Z-scores reaching as high as 35 in elbow flexion (Figure 13d), indicating substantial deviations from normal movement patterns. However, for shoulder flexion, Z-scores below 2 are still observed, suggesting that some abnormal movements might be misclassified as normal.

Finally, the Z-scores are converted into scores as shown in Equation (14). The results, shown in the last column of Figure 13, range from 0 to 100, with the highest scores concentrated in Subsets B and N, indicating that normal movements align closely with the baseline. Subset A (abnormal movements) shows more variation, with scores spread across a wider range. For shoulder flexion, the scores for abnormal movements fluctuate more than in other exercises, with the interquartile range spanning from 30 to 100. Although scores closer to 100 typically indicate normal movement, the broad range in shoulder flexion reflects greater variability in abnormal movements. This suggests that distinguishing abnormal movements is more challenging in exercises like shoulder flexion, where the line between normal and abnormal is less distinct. Overall, the scoring mechanism effectively quantifies deviations from the baseline but highlights the need for further refinement in classifying subtle abnormal movements.

**Figure 14:**
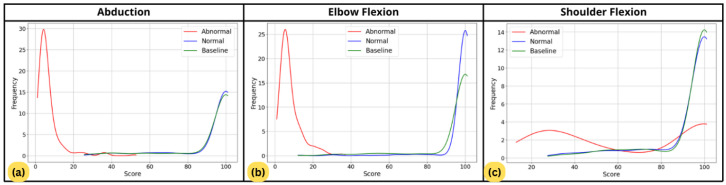
Score distributions for baseline, normal, and abnormal movements. (**a**) Abduction. (**b**) Elbow flexion. (**c**) Shoulder flexion.

In addition, Figure 14 shows the non-normal distribution curve of the scores, providing an alternative representation to the boxplots. It can be observed that for abduction and elbow flexion, the normal movements have scores mostly around 85, while the abnormal movements tend to have scores lower than 20. For shoulder flexion, although the normal scores remain centered around 85, the abnormal scores show more variation, ranging from below 60 to above 80.

In summary, the scoring mechanism effectively differentiates between normal and abnormal movements, with normal movements aligning closely with the baseline and abnormal movements showing more variation. The system performs well in exercises like abduction and elbow flexion but faces challenges in shoulder flexion, where the line between normal and abnormal is less distinct. Further refinement is needed for more accurate classification of subtle abnormal movements.

#### 4.2.4. Challenges in Shoulder Flexion Scoring

**Figure 15:**
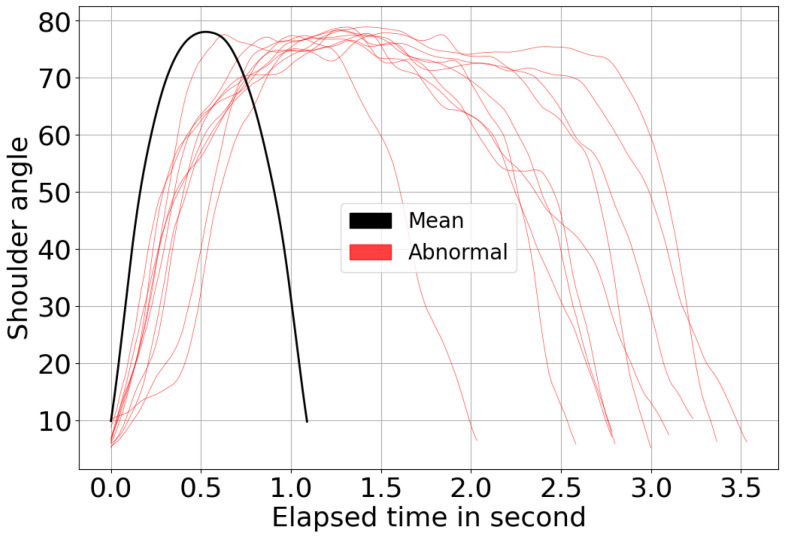
The 10 shoulder flexion motion segments (red) with the shortest distance to the baseline (black).

This subsection examines the challenges observed in shoulder flexion, which yielded the least favorable results. Figure 15 presents the 10 motion segments from Subset A (abnormal movement) with the shortest DTW distance to the mean trajectory of normal movement. Table 5 provides a statistical summary of these segments, including their mean and SD of distance, Z-score, and final score. Notably, all these segments received a perfect score.

One key factor influencing this outcome is that DTW focuses on spatial alignment while ignoring speed and timing variations. As a result, participants who executed the movement at a slower pace but still achieved the full range of motion were assigned scores comparable to those of normal subjects. This outcome aligns with rehabilitation principles, where achieving the target joint angles is more critical than movement speed. However, we expected that noised or inconsistent movements would result in lower scores. The fact that these irregularities were not sufficiently penalized suggests a limitation in DTW-based comparisons, highlighting the need for additional considerations—such as movement smoothness metrics or time-dependent features to improve classification accuracy.

Moreover, observations from recorded videos suggest that sitting posture affects the measured shoulder angle. Participants who leaned back against the chair exhibited lower shoulder flexion angles than those who sat upright, despite performing the same movement. This variation introduces inconsistencies in scoring and increases the challenge of distinguishing between normal and abnormal movements. The posture-related angle fluctuations in shoulder flexion contribute to higher variability compared to the other two exercises, making classification less reliable. Addressing these issues may require incorporating posture correction mechanisms or additional calibration steps to enhance assessment accuracy.

#### 4.2.5. Limitations and Concerns

While the scoring mechanism and methods presented in this study show promising results, there are a few limitations that need consideration. The evaluation was conducted using only three exercise motions, and the system’s performance may vary with different exercises or motion types. The abnormal movement data used in the experiment was simulated, and actual results from real patients or individuals with disabilities may differ due to the complex nature of their movement patterns. Additionally, the system was tested under controlled settings where motion performance and repetitions were clearly separated, which may not fully reflect real-world variability and challenges.

#### 4.2.6. Key Findings from Experiment

The evaluation demonstrated the effectiveness of the proposed DTW-based approach in classifying normal and abnormal movements. The system excelled at differentiating between normal and abnormal movements in exercises like abduction and elbow flexion, with clear distinctions observed. However, challenges arose in shoulder flexion, where the boundaries between normal and abnormal movements were less pronounced. This highlights the need for refinement, especially in exercises with subtle differences in movement patterns.

In terms of feature extraction, the single-angle method emerged as the most reliable for distinguishing between movement types, offering a simpler and more efficient solution compared to multi-angle approaches. The exploration of the scoring mechanism revealed its ability to quantify motor performance effectively, although some fine-tuning is required to improve the classification of subtle abnormal movements, particularly in exercises like Shoulder Flexion.

Overall, the system provides a solid foundation for differentiating normal and abnormal movements and quantifying deviations from baseline performance. However, further testing and refinement are necessary to enhance its accuracy and applicability across a broader range of exercises and patient populations.

## 5. Conclusions

In conclusion, this paper presents an innovative home-based exergaming system designed to facilitate motor recovery by combining engaging gameplay with standardized physical assessments. Unlike traditional exergames that either lack assessment or separate it from gameplay, our system integrates movement evaluation directly into the gaming experience. By leveraging MediaPipe for real-time pose estimation and DTW for movement comparison, the system evaluates users’ upper extremity movements based on the FMA-UE, providing real-time, actionable feedback.

Through the experimental results, we learned that the system effectively distinguishes between normal and abnormal movement patterns, demonstrating its potential for individualized monitoring of motor recovery. Notably, the system performed well in identifying deviations from normal movement, which is crucial for tracking progress and adjusting rehabilitation plans. However, we observed some challenges, particularly in exercises where the distinction between normal and abnormal movements was less clear, such as shoulder flexion. These insights suggest that while the system can accurately assess a range of movements, further refinement is needed to improve its sensitivity to subtle abnormalities.

The cloud-based nature of the system also allows caregivers to remotely monitor user progress on both PC and mobile devices, enhancing accessibility and facilitating more flexible rehabilitation management. This remote monitoring capability provides significant value, as it enables healthcare providers to adjust rehabilitation plans in real time, ensuring more personalized care.

Our findings underscore the potential of combining exergaming with physical assessments for motor recovery, providing a promising platform for home-based rehabilitation that encourages user engagement and improves adherence to therapy regimens. Future work could focus on refining the scoring system for more precise movement analysis, particularly in exercises with more nuanced movement dynamics, and exploring the long-term effects of this system on motor recovery outcomes. Expanding the system to include additional exercises and incorporating more sophisticated motion analysis techniques could further enhance its accuracy and effectiveness in real-world rehabilitation contexts.

## Figures and Tables

**Table 1:** Comparison of this work with exergaming systems for motor recovery across five dimensions: (A) Requires only a common webcam, (B) Independence from supervision, (C) Remote monitoring capability, (D) Integration of standardized physical assessments, and (E) Movement data collection or analysis during exergaming.

System	Year	A	B	C	D	E
Rabbit Chase [9]	2009	O	O	O	X	O
Tightrope Walk [15]	2015	X	X	X	X	O
Obstacle Avoidance [17]	2016	X	O	X	O	X
Motion Rehab AVE [16]	2017	X	X	X	X	O
Bowling [18]	2017	X	O	X	O	X
Crazy Target [22]	2021	O	O	O	O	X
Fish Frenzy [20]	2021	X	O	O	O	X
Ski Slalom [14]	2022	X	X	X	O	X
Airplane [19]	2022	X	O	O	X	X
Attack the Monster [21]	2022	O	O	O	X	O
Proposed System	2025	O	O	O	O	O

O: yes, X: no.

**Table 2:** *p*-values from Independent Samples *t*-test comparing pairs of data subsets for different feature extraction methods, including individual angles.

Exercise	Compared	Triple-Angle	Dual-Angle	Single-Angle	Shoulder	Elbow	Wrist
Abduction	Normal-Baseline	0.3242	0.8874	0.5555	0.5555	0.4563	0.6100
	Normal-Abnormal	0.0000	0.0000	0.0000	0.0000	0.0000	0.0000
	Baseline-Abnormal	0.0000	0.0000	0.0000	0.0000	0.0000	0.0000
Elbow flexion	Normal-Baseline	0.8606	0.8604	0.2109	0.9391	0.2109	0.7582
	Normal-Abnormal	0.0000	0.0000	0.0000	0.0540	0.0000	0.0000
	Baseline-Abnormal	0.0000	0.0000	0.0000	0.0437	0.0000	0.0000
Shoulder flexion	Normal-Baseline	0.0364	0.0672	0.6555	0.6555	0.1215	0.0977
	Normal-Abnormal	0.0000	0.0000	0.0000	0.0000	0.4608	0.0053
	Baseline-Abnormal	0.0000	0.0000	0.0000	0.0000	0.4010	0.0000

**Table 3:** *p*-values from Mann–Whitney U-test comparing pairs of data subsets for different feature extraction methods, including individual angles.

Exercise	Compared	Triple-Angle	Dual-Angle	Single-Angle	Shoulder	Elbow	Wrist
Abduction	Normal-Baseline	0.3468	0.9835	0.4619	0.4619	0.6806	0.5420
	Normal-Abnormal	0.0000	0.0000	0.0000	0.0000	0.0000	0.0000
	Baseline-Abnormal	0.0000	0.0000	0.0000	0.0000	0.0000	0.0000
Elbow flexion	Normal-Baseline	0.8992	0.7461	0.6022	0.8322	0.6022	0.9651
	Normal-Abnormal	0.0000	0.0000	0.0000	0.0000	0.0000	0.0000
	Baseline-Abnormal	0.0000	0.0000	0.0000	0.0000	0.0000	0.0000
Shoulder flexion	Normal-Baseline	0.0393	0.0753	0.7843	0.7843	0.0700	0.3451
	Normal-Abnormal	0.0000	0.0000	0.0000	0.0000	0.9745	0.0000
	Baseline-Abnormal	0.0000	0.0000	0.0000	0.0000	0.0854	0.0000

**Table 4:** Mean ($\bar{x}$) and standard deviation (*s*) of distance values calculated using the single-angle method.

	Baseline	Normal	Abnormal
Abduction	$\bar{x}$: 0.8348	$\bar{x}$: 0.8144	$\bar{x}$: 9.2378
	*s*: 0.3862	*s*: 0.3735	*s*: 3.9171
Elbow flexion	$\bar{x}$: 1.4478	$\bar{x}$: 1.3737	$\bar{x}$: 13.9217
	*s*: 0.6973	*s*: 0.5932	*s*: 7.2972
Shoulder flexion	$\bar{x}$: 2.0120	$\bar{x}$: 2.0726	$\bar{x}$: 4.5952
	*s*: 1.4640	*s*: 1.5109	*s*: 3.1200

**Table 5:** Statistical summary for the closest 10 abnormal shoulder flexion distances.

	Mean	SD
Distance	1.9940	0.0532
Z-Score	−0.0123	0.0363
Score	100	0

## Data Availability

The source code used for motion segmentation and analysis in this study is openly available at the following GitHub repository: https://github.com/ArmNorapat/norapat-motion-segmentation (accessed on 20 October 2025). Due to privacy restrictions involving human participant video recordings, the movement data collected during the experiment are not publicly available. An anonymized subset of the data may be provided upon reasonable request to the corresponding author.

## Abbreviations

The following abbreviations are used in this manuscript:

DTW	Dynamic Time Warping
FMA-UE	Fugl–Meyer Assessment for Upper Extremity
MDM	Motion Detection Module
PAM	Physical Assessment Module
RM	Report Module
ROM	Range of Motion
WMFT	Wolf Motor Function Test
BBT	Box and Block Test
DDA	Dynamic Difficulty Adjustment
